# A Water-Soluble Microencapsulated Milk Thistle Extract as Active Ingredient for Dermal Formulations

**DOI:** 10.3390/molecules24081547

**Published:** 2019-04-19

**Authors:** Tiziana Esposito, Francesca Sansone, Paola Russo, Patrizia Picerno, Rita Patrizia Aquino, Franco Gasparri, Teresa Mencherini

**Affiliations:** Department of Pharmacy, University of Salerno, Via Giovanni Paolo II, 132, 84084 Fisciano (SA), Italy; tesposito@unisa.it (T.E.); paorusso@unisa.it (P.R.); ppicerno@unisa.it (P.P.); aquinorp@unisa.it (R.P.A.); info@gasparrfranco.it (F.G.); tmencherini@unisa.it (T.M.)

**Keywords:** silymarin, delivery system, hydrogel and emulgel, lecithin, stability and in vitro permeation profile

## Abstract

The choice of formulation is often of crucial importance in order to obtain a pharmaceutical product for the administration of poorly soluble drugs. Recently, a new water-soluble microparticulate powder form (MTE-mp) for the oral administration of a high functionality/low solubility silymarin rich milk thistle extract (MTE) has been developed. Findings showed that extract-loaded microparticles by spray-drying were produced with high and reproducible yields and encapsulation efficiency. The in vitro dissolution and permeation rates of silymarin were dramatically improved with respect to the raw material, and also enhanced the silymarin anti-inflammatory abilities. Given these successful results, the new MTE-mp delivery system has been proposed as an active ingredient for dermal applications. The aim of this research was the design and development of two topical formulations, hydrogel and emulgel (O/W emulsion), containing the MTE-mp delivery system or MTE raw extract. All the formulations were compared to each other in terms of handling and incorporation amount of the active ingredient during the productive process. Moreover, the addition to the emulgel of lecithin (L) as enhancer of permeation was tested. The MTE-mp ingredient that resulted was stable and more-easily incorporated both in hydrogel and emulgel than raw MTE extract, obtaining the best permeation profile for MTE-mp from emulgel with the addition of L. The obtained results confirm that the MTE-mp system could be used as a stable, water-soluble, and easy-handling functional ingredient, giving the opportunity to develop new strategies for MTE delivery in health products.

## 1. Introduction

Milk thistle (*Silybum marianum* (L.) Gaertn. (Asteraceae)) extract (MTE) is an important source of silymarin. It is a well-known complex of flavonolignans (silybin A and B, isosilybin A and B, silydianin, and silychristin) and flavonoids (taxifolin and quercetin) used to reduce inflammation, also having membrane-stabilizing, hepatoprotective, anticarcinogenic, and antiviral activities [1]. MTE extract is noted to be able to decrease cellular peroxidation and protect skin from photo aging, as well as ultraviolet rays UVB-induced damages and carcinogenesis [2,3]. Further studies have already shown that topical application of silymarin suppresses intracellular production of hydrogen peroxide, and nitric oxide and reduces depletion of catalase in UVB-irradiated mouse skin [4,5]. Unfortunately, MTE clinical use is negatively influenced by low aqueous solubility, which limits oral and topical administration [6]. Moreover, the silymarin complex and its main component, silybin, which is well known for its broad spectrum of biological properties, [7,8], react readily with various environmental factors and undergo to oxidation/degradation process with consequent functionality decrease. The recommendations of the manufacturer to store silybin standards are to keep them at 4 °C and away from light, to avoid degradation. To overcome these limitations, we recently produced a new stable water-soluble microparticulate powder system using spray drying technology containing a silymarin-rich extract (MTE-mp) [9]. MTE-loaded microparticles by spray drying were produced with high and reproducible yields and encapsulation efficiency. NaCMC, used as a coating/swelling polymer in a proper solvent system, was able to protect the functionality of the extract over time. The in vitro dissolution and permeation rates of silymarin were dramatically improved suggesting a higher bioavailability after the administration [9,10]. Silymarin anti-inflammatory abilities were preserved by the encapsulation process, as demonstrated by immune cell exposure to MTE-mp and inflammatory cytokine suppression, which suggest that the microencapsulation process didn’t affect silymarin efficiency.

It has been demonstrated that topical administration of silymarin complex provides an efficient way to enrich the endogenous cutaneous protection system, and thus may be a successful strategy for diminishing ultraviolet radiation-mediated oxidative damage in the skin [2,3,11]. Recently, much research has been focused on the potential use of this drug as a free-radical scavenger to prevent oxidative skin damage, and its topical application has been met with considerable interest. Recently, a binary complex composed of MTE and beta-cyclodextrins was used to well functionalize emulsions designed for skin delivery, and to enhance the silymarin permeation rate [12]. Based on these evidences, the aim of the present research was to develop two functional topical formulations to support MTE-mp potential use as active ingredient in formulation for skin delivery. Milk Thistle extract loaded microparticles were used to enrich two topical formulations: a hydrogel, to evaluate the ability of MTE-mp to be easily enclosed in a monophasic formulation, and an emulgel, in which the oil in water (O/W) emulsion was stabilized with the same gelling agent of the hydrogel as a delivery system. As a result, the emulgel possessed the characteristics of both emulsion and gel, which made it a dual control release system [13,14]. Also, it was proven to be the most convenient and effective topical delivery system, especially for hydrophobic drugs, and was commonly used for the treatments of skin disorders [15,16].

A technological characterization of the MTE-mp-loaded hydrogel and emulgel, with respect to the MTE raw extract, was performed on loaded and blank (without extract) microparticles. The products were characterized in term of stability (centrifuge test), dimensional distribution (by laser light scattering, LLS), and distribution of active ingredient (by fluorescence microscopy, FM). Moreover, the main chemical–physical characteristics (pH, viscosity) of all produced formulations were analyzed. Given the different natures (hydrogel and emulgel), a specific method was devised to evaluate the MTE-mp permeability from each type of formulation through synthetic membranes by means of Franz Cells. During recent years, some authors [17] have proposed the use of substances endowed with low toxicity (i.e., phospholipids) as penetration enhancers [18]. These substances present a notable affinity with cellular membranes, thus leading to an increased absorption of several drugs, and also improving the fluidity of the lipid formulation phase [19]. Thus, in our study, soy lecithin (L) was added to the emulgel formulations to enhance the in vitro permeation properties with a potential optimization of the incorporated MTE bioavailability.

## 2. Results and Discussion

### 2.1. Preparation of Hydrogel and Emulgel

The compositions of all prepared formulations are reported in (Section 3. The preparation methods are described in the Section 3.2. The choice to select two different formulations was done in order to verify how their composition and physical characteristics are able to influence the administration of the active ingredients studied. During the gel manufacturing, the MTE-mp ingredient was more-easily incorporated into the aqueous system than the raw MTE, obtaining a homogeneous distribution of the active ingredient with less mechanical agitation. An emulsion that is more complex in numbers and types of ingredients, with a more complex production process, is nonetheless preferred for cosmetic and pharmaceutical formulations for the simultaneous incorporation and delivery of hydrophilic and lipophilic active ingredients to the skin. Moreover, the biphasic system (oil, water) is more appealing to the application, and able to mask the disagreeable taste and odor of some ingredients [20]. Thus, the raw MTE and MTE-mp were separately added to an oil-in-water (O/W) cosmetic emulgel. A very difficult and incomplete incorporation was obtained for the MTE emulgel, probably due to the crystalline solid state and low solubility of the raw material. Otherwise, MTE-mp, due to the microparticulate amorphous solid state, was easily incorporated into the emulgel up to 6% *w*/*w*. Generally, the addition of plant extracts at high concentrations can compromise the organoleptic characteristics and the physical-chemical stability of an emulsion [21], and thus it is necessary to verify the adequacy of the MTE- and MTE-mp-loaded emulgel, as well as to compare the influence of each active ingredient on the formulation properties.

### 2.2. Macroscopic Analysis and Stability of Emulgel Formulations

**Figure 1:**
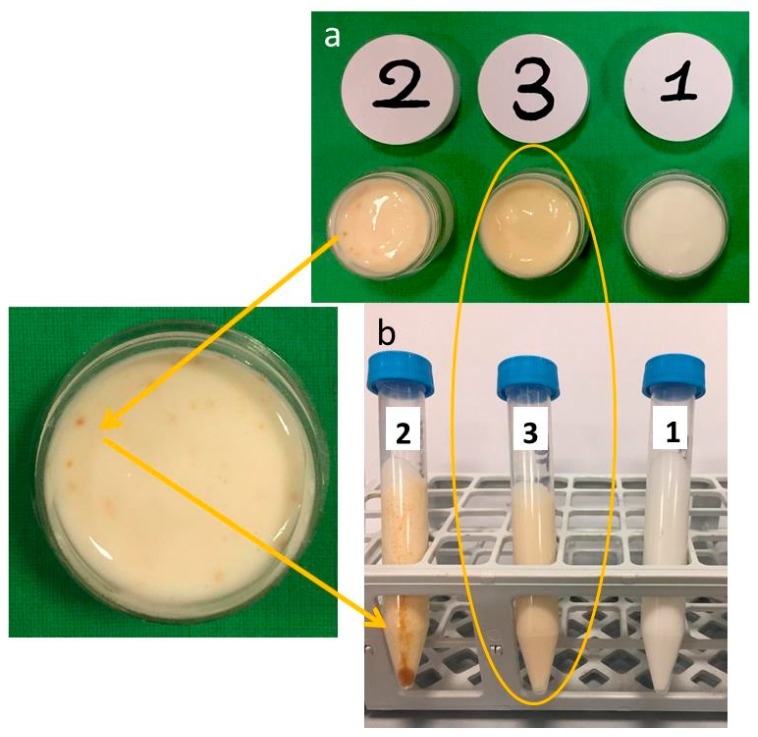
Macroscopic appearance of emulgel formulations before (**a**) and after (**b**) the centrifuge test (1 = blank emulgel; 2 = raw Milk Thistle Extract (MTE) emulgel; 3 = MTE-mp emulgel).

As expected, the addition of plant extract to both raw MTE (Figure 1a no. 2) and MTE-mp (Figure 1a no. 3) changed the color of the blank emulgel (Figure 1a no. 1) from white to pale yellow (nos. 3 and 2). MTE, however, was not completely solubilized, resulting in a non-homogeneous distribution in the biphasic system, as demonstrated by the presence of brown agglomerates of the extract in emulgel no. 2 (Figure 1a).

On the contrary, the color of the MTE-mp emulgel (no. 3) appeared uniform, suggesting a good solubilization and distribution of the active ingredient.

The conclusions of the visual (macroscopic) examination were confirmed by a centrifuge test, performed with two cycles, at 4000 rpm for 30 min and 5300 rpm for 15 min, on the same sample. The test results are depicted in Figure 1b. The emulgel no. 2 (with raw MTE) showed a phase breaking as well as clear precipitation (brown pellet) and creaming (brown aggregates) of MTE. Otherwise, the MTE-mp-loaded emulgel (Figure 1b no. 3) is stable after the test, such as the blank emulgel (Figure 1b no. 1). In particular, the loading of the emulgel with a high amount of MTE-mp (6% *w/w*) did not affect the stability of the polymeric network system, while it did obtain a homogeneous distribution of the actives.

The pH value of dermal formulations must be compatible with the skin at the application (pH range 4.0–6.0). A product with a high-alkaline pH could favor the proliferation of pathogenic bacteria, while a high-acid pH could destroy the bacterial flora that physiologically protects the skin surface. The importance of an adequate regulation of pH also lies with effectiveness of preservatives in a narrow range of acid values, while they lose activity at alkaline values favoring the microbiological instability of formulation [22]. The pH values were in an adequate range for all the produced formulations (Table 1).

Viscosity plays an important role in controlling drug permeation, and was an important parameter for evaluating the physical stability of the formulation. Hydrogel showed higher values of viscosity than emulgel (Table 1); the higher viscosity values detected for the MTE-mp-loaded formulations compared with the other ones was probably due to the presence of the functional ingredient sodium carboxymethyl cellulose (NaCMC) in the MTE-mp, as it contributed to the increase in the viscosity of the system.

### 2.3. Morphological Analysis of Emulgel Formulations

**Figure 2:**
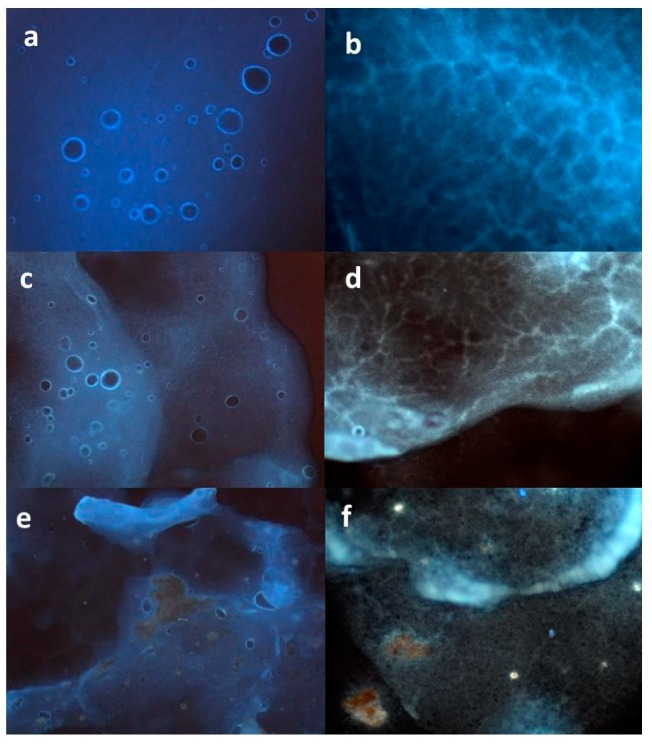
Fluorescence microphotographs of emulgel formulations (**a**,**b** = blank emulgel; **c**,**d** = MTE-mp emulgel; **e**,**f** = raw MTE emulgel).

The characterization by microscopic techniques is essential to obtain reliable data about the actual morphology of the system. In a previous work, an original morphological analysis was conducted by fluorescence microscopy (FM) [23] to verify the distribution of an active extract into an O/W emulsion system. The same method has been applied here to evaluate differences in the homogeneity of MTE or MTE-mp distribution into the emulgel system [9]. The blank formulation resulted well-structured, showing a homogeneous distribution of spherical oil droplets in the aqueous phase. This was analyzed by imaging the superficial area (Figure 2a), which was found to also have a network-formed internal structure (Figure 2b) that was able to confer physical stability to the formulation.

There were critical differences between the MTE- and MTE-mp-loaded emulgels. Figure 2c shows the MTE-mp emulgel with the same structure as the blank emulgel, with augmented fluorescence and a well-dispersed presence of the extract within the aqueous phase and around the oil droplets. Moreover, the presence of the MTE-mp ingredient did not affect the integrity of the internal polymeric network (Figure 2d). The homogeneity in the distribution was probably due to the microparticulate form of the technological ingredient, which appropriately interacted with the emulgel components. Otherwise, the raw MTE emulgel showed an incomplete interaction between the active ingredient MTE and the emulsion system. The MTE extract was partially visible, as crystals not incorporated in the droplet phase also negatively affected the superficial and internal properties of the emulgel system (Figure 2e,f).

### 2.4. Dimensional Analysis of Emulgel Formulations

**Figure 3:**
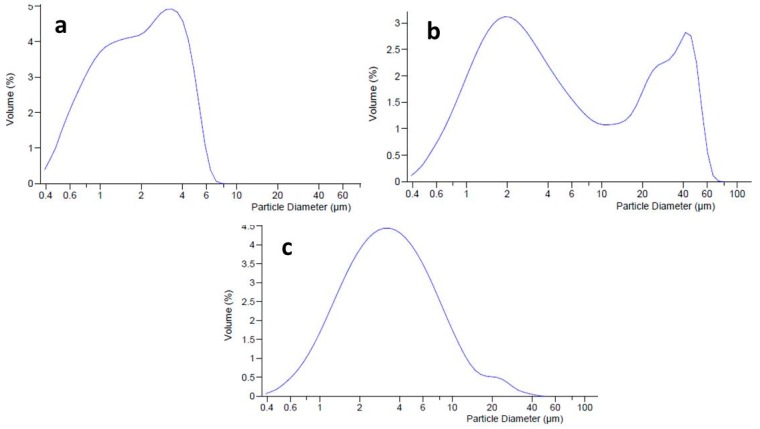
Dimensional distribution of blank emulgel (**a**), MTE emulgel (**b**), and MTE-mp emulgel (**c**).

Dimensional analysis (by LLS) was conducted to evaluate the distribution of oil droplets into the internal phase of the emulgel, and the effects of the active ingredients MTE and MTE-mp on the dimensional distribution of the emulgel system. The unloaded (blank) emulgel (Figure 3a) had a homogeneous dimensional distribution with an average diameter (d_50_) of dispersed phase droplets of 2.488 µm (span value 1.14). It was evaluated that loading the emulgel with raw MTE influences the size distribution of the dispersed phase. Graphically, a bimodal trend of the distribution curve was visible (Figure 3b), which was caused by the presence of two different dimensional populations. This result can be attributed to the coexistence of the dispersed phase droplets not being adequately incorporated in the formulation with the extract. The average diameter of droplets was in the micrometric range (d_50_ 6.045 µm), but the span value, due to the bimodal behavior, was very high, confirming the inhomogeneity of the formulation (span value 11.45; d_90_ 39.55 µm) [24]. The loading of MTE-mp at 6% *w/w* did not particularly influence the size distribution of the emulgel, which had a slightly larger dispersed phase (d_50_ 3.281 µm) than the unloaded one but with a practically unimodal size distribution (Figure 3c). This result could be due to the different solid states of both materials. As previously reported, raw MTE extract showed a crystalline solid state with a dimensional distribution of about 20 µm with respect to the MTE-mp, which had a particulate powder form in an amorphous solid state with a smaller size distribution of about 4 µm [9]. As a matter of fact, the microencapsulated extract MTE-mp was suitable to be adequately incorporated in the formulation.

### 2.5. In Vitro Permeation Study: Hydrogel vs Emulgel

Skin damage is often treated topically with active ingredients dissolved in gel or emulgel vehicles. Permeation of active ingredients through the epidermis is a prerequisite for an effective topical formulation. A synthetic, non-animal-based model of an artificial membrane, predictive of diffusion in human skin and able to mime in vitro the physiological cutaneous barrier, was used for transdermal diffusion testing to study the rate of MTE release from the produced formulations (hydrogel and emulgel). The aim of this part of the work was to evaluate a potential dual behavior regarding the permeation rate of MTE due to the different formulations, membranes, used mediums, and active ingredient form (MTE raw material or MTE microparticles).

**Figure 4:**
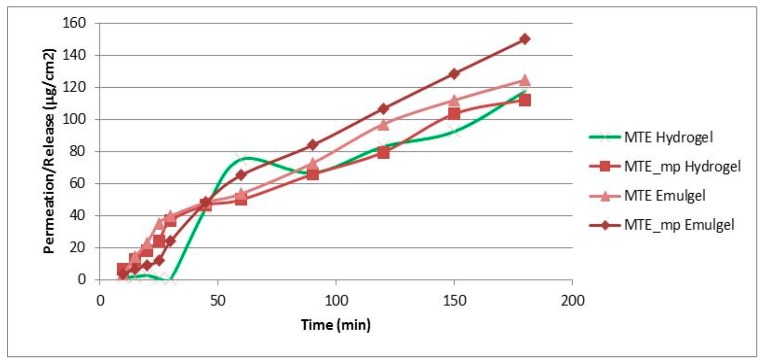
In vitro permeation release study through Franz Cells of all produced formulations (standard deviation for each point < 1%).

The results of the MTE and MTE-mp permeation experiments from the hydrogel and emulgel are shown in Figure 4 as the amount of permeate extract/permeation area as a function of the time elapsed.

The permeation profiles show that MTE (green and pink lines) permeated in a lower quantity than MTE-mp (lines with square and rhombus), and that the time release of MTE from the hydrogel (green line) was inconstant and non-linear. On the contrary, the microencapsulated MTE-mp permeated in greater quantity and in a constant manner over time. The observed behavior can be explained both on the basis of the different water dissolution rates and permeation properties of the MTE raw material and MTE-mp technological ingredient when applied as pure products [9], as well as on the basis of the previously made observations about the formulation and incorporation of the two powders in the hydrogel. In fact, MTE that presented a low dissolution rate, low permeation [9], and non-homogeneous dispersion into the hydrogel, had a lower permeation rate and a non-constant release profile from the formulation; MTE-mp, which is a technologically improved ingredient, had a higher dissolution rate and greater permeation [9], and was homogeneously incorporated into the hydrogel, also showing a greater permeation with a linear release profile.

The permeation studies carried out on the emulgel (pink line and rhombus) showed that, even in this case, MTE permeated in a lower quantity than the microencapsulated MTE-mp.

However, the type of formulation influenced the permeation properties; in fact, the permeation percentage obtained by the emulgels with the same extract (raw or microencapsulated) were clearly greater than the permeation of the same products by hydrogel. These results led us to select the MTE-mp emulgel as the formulation to be optimized for the topical delivery of milk thistle extract.

### 2.6. Optimization and Technological Analysis of the Emulgel

#### 2.6.1. Addition of Lecithin (L) as Permeation Enhancer

Suitable percutaneous absorption is known to be an essential requirement for satisfactory topically applied photo-protective agents such as silymarin complex, and may be improved by selecting the appropriate penetration enhancers.

Penetration enhancers may be present in topical and transdermal systems in order to increase skin permeability to drugs, as well as to influence the physical properties of semi-solid preparations.

As reported elsewhere [17,25], the presence of enhancers of dissolution rates and skin penetration such as the lecithin (L) influence release profile, permeation through membranes, and skin retention of a lipophilic drug, also positively affecting the photoprotective effect of some phenolic acids [26].

Based on these evidences, the influence of added L on the formulation characteristics and in vitro permeation properties of MTE-mp from the emulgel was investigated.

L was added to the formulation when the aqueous phase reached 65 °C, stirring until complete dissolution.

#### 2.6.2. Stability and Macroscopic Analysis of L-Emulgel Systems

The presence of L led to a decrease in the viscosity of both the formulations (L-blank and L-MTE-mp emulgel) (Table 2), compared to the formulations produced without L. The viscosity reduction did not affect the stability of the emulgel, but it could be an advantageous technological characteristic in terms of the release of the extract after dermal application [13].

The pH of the formulations remained dermo-compatible (range between 5.32 and 5.52), and comparable with the values of the emulgels without L (Table 1).

#### 2.6.3. Dimensional Analysis of L-Emulgel Systems

**Figure 5:**
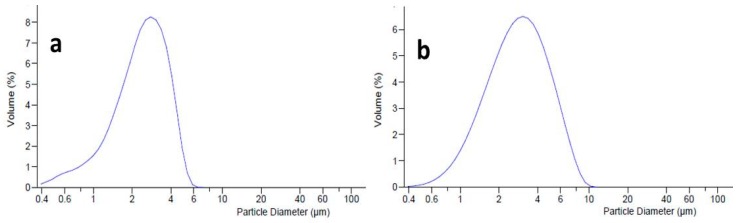
Dimensional distribution curve of L-blank emulgel (**a**) and L-MTE-mp emulgel (**b**).

The presence of L in the emulgel (L-blank emulgel, Figure 5a) reduced the size distribution of the droplets (d_50_ 1.935 µm) in the oil phase, producing a more homogeneous dispersion than the emulgel formulated without L and positively affecting the stability of the system [27].

For the MTE-mp emulgel (L-MTE-mp emulgel, Figure 5b), the addition of L improved the dimensional distribution behavior both in terms of reduction of the average diameter (d_50_ of 2.626 µm) and in homogeneity. This result is graphically expressed by the loss of the slight tailing of the distribution curve, and by the lowest value of the span (2.3) obtained.

#### 2.6.4. In Vitro Permeation Study: Effect of Lecithin

**Figure 6:**
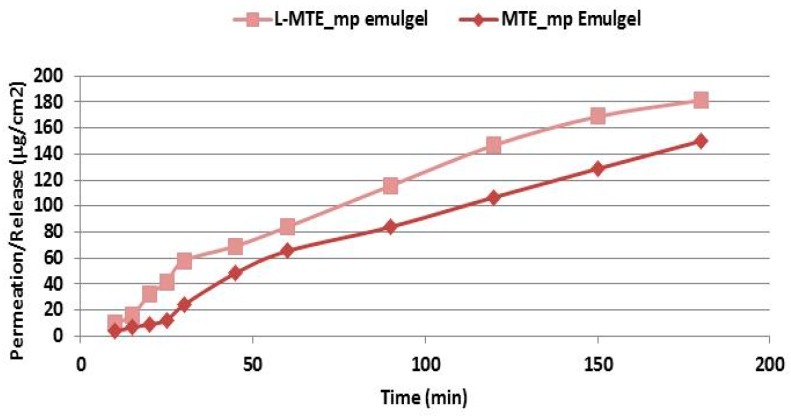
Permeation profile of MTE-mp emulgel with (pink line) or without L (line with rhombus) (standard deviation for each point < 1%).

The results of the MTE-mp permeation in vitro experiments using the emulgels with and without L are shown in Figure 6, and expressed as the amount of permeate extract/permeation area as a function of the time elapsed. As reported in commercial product data sheet, the synthetic membrane used, Strat-M, is declared to exhibit differential permeability in the presence of enhancers and to be compatible with common formulation formats, making it appropriate for use during formulation optimization.

As is evident from the results reported in the graph, the permeation of MTE-mp improved further using the emulgel with L. This behavior could be due to the interaction that L, with its flexible and phospholipid-like structure, has with the lipid ingredients of the emulgel, which normally tend to retain the extract in formulation. The presence of L probably facilitates the release of the extract, making the structure of the internal lipid phase of the emulsion more fluid, and favoring the exposure of the extract to the receiving compartment [28]. Since it is a passage through a synthetic and non-biological membrane, it is not possible to evaluate the effect that L would have as a membrane-perturbing agent.

## 3. Experimental

### 3.1. Materials

Hydrogel was obtained using methylpropanediol, caprylyl glycol, phenylpropanol, MTE, arginine, hydrogenated lecithin, acrylates/C10-30 alkyl acrylate crosspolymer, kindly provided by MedaPharma/Rottapharm (Madaus spa, Monza, Italy).

Emulgel was obtained using cetearyl alcohol, caprylic/capric triglyceride, cimethicone, glyceryl laurate, steareth-21, steareth-2, polypropylene glycol -15 stearyl ether, butylated hydroxytoluene, butyrospermum parkii, hydrogenated lecithin, methylpropanediol, caprylyl glycol, phenylpropanol, arylates/C10-30 alkyl acrylate crosspolymer, arginine from MedaPharma/Rottapharm (Madaus spa, Monza, Italy). MTE-mp powders were obtained as described by Sansone et al. [9]. Other reagents used are all of pure grade.

### 3.2. Dermal Formulations Preparation

The unloaded formulations were coded as blank, and with lecithin as L-blank. The loaded formulations with raw MTE extract were coded as MTE, and the loaded formulations with MTE microparticles were coded as MTE-mp and L-MTE-mp. All the formulations were compared with each other in terms of stability and their primary chemical–physical characteristics (pH, viscosity).

#### 3.2.1. Hydrogel Preparation

The composition of hydrogels (blank, MTE, and MTE-mp) are reported in Table 3. The hydrogels were prepared by heating the water to 65 °C, then adding the acrylates/C10-30 alkyl acrylate crosspolymer and weighing separately. The polymer was hydrated for 15 min at room temperature, then methylpropanediol, caprylyl glycol and phenylpropanol were added. The raw extract (MTE) and MTE microparticles (MTE-mp) were separately added at room temperature, under stirring (2 min for the MTE-mp and 10 min for the raw MTE extract). Finally, arginine was added up to pH 5.5, stirring until the hydrogel was formed.

#### 3.2.2. Emulgel Preparation

The emulgel was an O/W emulsion stabilized with the same gelling agent as the hydrogel. Table 4 reports each component of the emulgel formulation. The oil phase (cetearyl alcohol, caprylic/capric triglyceride, dimethicone, steareth-21, steareth-2, polypropylene glycol -15 stearyl ether, butylated hydroxytoluene, Butyrospermum parkii, and lecithin), was heated all together at 65 °C; except for glyceryl laurate was added once the final temperature was reached. The aqueous phase (methylpropanediol, caprylyl glycol, phenylpropanol, aqua, and acrylates/C10-30 alkyl acrylate crosspolymer) was separately heated at 65 °C. The oil phase was added to the aqueous one under mechanical agitation by Silverson model SL2T (CRAMI Group srl, Milano, Italy), at 8000 rpm for 10 min. The obtained emulgels were manually stirred until 35 °C. The raw MTE extract and MTE microparticulate (MTE-mp) were slowly introduced, at room temperature, into the emulgels. Finally, arginine was added up to pH 5.55. A formulation without the active ingredient (blank emulgel) was prepared with the same procedure and used as the control.

Due to the content of 50% of extract in MTE microparticles (50%) [9], the MTE-mp ingredient has been added doubled the amount of MTE raw extract, to obtain the same loading of extract for MTE and MTE-mp formulations. 

### 3.3. Viscosity, pH, and Stability Studies by Centrifuge Test

#### 3.3.1. Centrifuge Test

We placed 7 g of emulgel in a graduated tube for a centrifugation test carried out at 25 °C using a Labofuge 200 Centrifuge (Thermo Scientific, Waltham, MA, USA). The test was performed at 4000 and 5300 rpm for 30 and 15 min, respectively. The phase separation and other instability phenomena were evaluated for macroscopic evidences.

#### 3.3.2. pH Determination

The pH was measured with a digital pH meter (MP220, Mettler Toledo, Columbus, OH, USA) at 22 ± 2 °C.

#### 3.3.3. Viscosity Evaluation

The viscosity measurement was carried out with a Visco Basic Plus viscometer (FungiLab S.A., Barcelona, Spain) at 25 °C, using a Spindle L4 at 12 rpm (for hydrogel) and L4 at 3 rpm (for emulgel). Each reading was done for 10 and 20 s, respectively, after the rotation speed started. The results were expressed as centipoise (cP).

The percentage error values were detected in relation to the confidence limit of the instrument and the number of analyses performed. All the values obtained with a percentage error greater than 2% were discarded.

### 3.4. Dimensional Analysis of Emulgel

The size distribution of the droplets constituting the dispersed phase of the formulations was evaluated by means of liquid module laser scattering diffraction (LS 13 230 Particle Volume Module Plus Beckman Coulter Inc, Brea, CA, USA). The samples were prepared by diluting the formulations in deionized water; a few drops of each sample were then introduced into the analysis cell so as to obtain an optimal darkening [29] between 8% and 12%. The dimensional distribution of the powders was then processed with a specific software using the Fraunhofer mathematical model. The analyses were carried out in triplicate: the results were expressed as d_50_ (average diameter) and as span [(d_90_–d_10_)/d_50_], a parameter that takes into account the polydispersity of the droplet size distribution in the dispersed phase; the higher the span value, the greater the inhomogeneity of the size distribution.

### 3.5. Morphological Analysis

Morphology of the blank and MTE- and MTE-mp-loaded emulgels was examined by fluorescent microscopy (FM). The samples were spread on a slide and dried under inert nitrogen flow. The dried sample was imaged by FM using a Zeiss Axiophot fluorescence microscope, with a 2.5 and 20x ± 1.4 NA plan apochromat oil immersion objectives (Carl Zeiss Vision, München–Hallbergmoos, Germany) using standard DAPI (40,6-diamidino-2-phenylindole) optics that adsorb violet radiation (max. 372 nm) and emit a blue fluorescence (max. 456 nm).

### 3.6. In Vitro Permeation Study

#### 3.6.1. Selection of Solvents for Receiving Compartment

Preliminary experiments indicated that the use of water was adequate to evaluate the permeation profiles of the tested extract only from the hydrogel formulation, but not from the emulgel. In the latter case, the components of the oil phase (silicones, butters, and consistency factors) partially permeated together with the extract, causing problems both with occlusion of the membrane pores and in the column during the subsequent quantitative analysis by reverse phase high-performance liquid chromatography-photodiode array detector RPHPLC–DAD. For this reason, in a second set of experiments, a different receiving compartment was selected that was adequate to analyze both formulations in parallel. In particular, literature data [30,31] indicate that appropriate amounts of organic solvent in the receiving compartment can facilitate the analysis of samples containing fat and/or water-soluble phases; therefore, different hydro alcoholic solutions (water/ethanol in different ratios) were tested for the evaluation of the in vitro permeation of the systems under examination. The study of the methods reported in the literature, as well as the assessment of the adaptability of these methods to the formulations and extracts under examination and to the chromatographic technique for the quantification of the sample, allowed us to select a hydro-alcoholic solution composed of ethanol (EtOH):water ratio of 70:30 as a receiving compartment.

#### 3.6.2. Test Set Up

The experiments were conducted according to [23] with slight modifications. The release/permeation behavior of MTE from the formulation was measured in Franz-type vertical diffusion cells (Hanson research corporation, HansonResearchCorporation, Chatsworth, CA, USA) with permeation areas of 1.7671 cm^2^, applying a SKIN STRAT–TM^®^ membrane between the donor and receiving compartment, and assuring the absence of air bubbles. Strat-M membrane is suitable for transdermal diffusion testing; it is a synthetic, non-animal based model for transdermal diffusion testing that is predictive of diffusion in human skin, and is designed for screening of active pharmaceutical ingredients (API), cosmetic actives, formulations, personal care products, pesticides, and chemicals. Each cell was filled with 7 mL of previously degassed receptor fluid (EtOH:water, 70:30) and heated by a thermostatic re-circulating water bath at 37 °C. During the experiments (up to 3 h), the receptor fluid was continuously stirred (170 rpm) by magnetic stirring. Prior to the application of the test sample, the system was stabilized for 15 min. In order to increase the volume of the donor compartment, three dosage wafers (each of 15 mm diameter and 1 mm height) were stacked together. Thus, 1 g of MTE or MTE-mp-loaded formulation was uniformly applied on the membrane surface and subsequently sealed with spring clips and laboratory Parafilm. At appropriate time intervals, aliquots of the receptor fluid (200 µL) were taken and immediately replaced by the fresh receptor mixture. The samples were filtered and directly analyzed by HPLC–DAD for the determination of silybin used as a marker of MTE, as above reported. The amount of MTE permeated per area (Q) for each time interval was calculated by means of the following equation:

$$Q\cdot (\frac{mg}{cm^{2}})=\frac{V_{R}\times C_{N}+\sum_{i=n}^{n-1}V_{P}\times C_{i}}{A}$$

where *V_R_* is the receiver volume, *C_N_* is silybin concentration in the receiver at the time *n*, *V_P_* is the volume of the removed sample, and *C_i_* is the silybin concentration in the receiver at the time *n* − 1. Permeation data were reported as the quantity of permeated silybin per permeation area related to time. All the permeation tests were made in triplicate; only the mean values are reported (standard deviations for each point <1%).

#### 3.6.3. Quantitative Analysis

For the quantitative determination of the main active silybin, it was necessary to first determine the calibration curve of silybin in the solvent ratio of EtOH:water 70:30. *Linearity.* Silybin reference standard solutions were prepared at three concentration levels (100–400 µg/mL) and injected three times. The standard curve was analyzed using the linear least-squares regression equation derived from the peak area (regression equation *y* = 2915.3*x* − 63.725, *r^2^* = 0.9998, where *y* is the peak area and *x* the concentration). Silybin content was evaluated by an HPLC apparatus (Agilent 1100 series system equipped with a Model G-1312 pump, a Rheodyne Model G-1322A loop (20 μL), a DAD G-1315 detector, and a 150 × 3.9 mm i.d. C-18 μ-Bondapack column). Peak areas were calculated with an Agilent integrator. The solvents were HCOOH 0.1% in H_2_O (solvent A) and HCOOH 0.1% in MeOH (solvent B). Elution gradient: 0 → 5 min, 43 → 45% B; 5 → 10 min, 45% B; 10 → 115 min, 45 → 50% B. Analysis was carried out in triplicate: flow rate of 0.8 mL/min; DAD detector set at 288 nm. *Specificity.* Peak associated with the marker was identified by its retention time (t_r_ = 26.12 min) and confirmed by co-injection with a standard.

## 4. Conclusions

Flavonolignans of silymarin complex extracted from milk thistle could act as chemopreventive or therapeutic adjuvants, having hepatoprotectant, anti-inflammatory activities, as well as being able to protect skin from photoaging. Milk thistle extract clinical use is negatively influenced by its low stability and aqueous solubility which limits oral and topical administration. The loading of MTE into sodium carboxymethyl cellulose microparticles (MTE-mp) by spray drying technique was able to overcome these critical characteristics, enhancing the bioavailability of functional silymarin complex after the intake. The engineering microparticulate form of MTE was used as an active ingredient to functionalize two topical formulations, hydrogel and emulgel. The MTE-mp technological ingredient was more easily incorporated in both formulations than the raw extract. In particular, the loading of emulgel with a high amount of MTE-mp (6% *w/w*), did not affect the stability of the polymeric network system, and also obtained a homogeneous distribution of the active ingredients. The optimization of the emulgel with enrichment by lecithin (L-MTE-mp emulgel) as a permeation enhancer reduced the dimensional distribution of oil droplets, making the structure of the internal lipid phase of the emulgel more fluid. The good interaction between lecithin and MTE-mp into the emulgel system facilitated the release of the active ingredients from the formulation, favoring exposure of the extract to the receiving compartment and improving the in vitro skin permeation. These results can represent the basis for future trials to validate the in vivo efficiency of MTE-mp as a functional, easy-handling ingredient to be used as a therapeutic agent for the treatment of skin damages.

## Figures and Tables

**Table 1:** pH and viscosity values of produced hydrogels and emulgels.

**Hydrogel**	**pH**			Viscosity (centi Poise-cP) **		
	t_24h_ *	**t_48h_**	t_30d_ *	**t_24h_**	**t_48h_**	**t_30d_**
Blank	5.12	5.11	5.02	30328	27715	27373
MTE	5.15	5.14	5.09	25567	23584	25734
MTE-mp	5.24	5.21	5.17	32141	32194	31539
**Emulgel**	**pH**			Viscosity (cP) ***		
Blank	5.31	5.30	5.29	138534	117118	181784
MTE	5.22	5.20	5.25	121479	162614	137004
MTE-mp	5.42	5.40	5.49	195952	191645	211094

* h = hours; d = days; ** L4, 12 rpm 10’’; *** L4, 3 rpm 20’’.

**Table 2:** pH and viscosity values of emulgels produced with lecithin.

Emulgel	pH			Viscosity (cP) **		
	t_24h_	t_48h_	30_d_ *	t_24h_	t_48h_	30_d_
^+++^L-blank	5.32	5.49	5.52	124594	93769	105306
L-MTE-mp	5.42	5.33	5.49	122318	112383	94559

* h = hours; d = days; ** L4, 3 rpm 20’’; ^+++^ L = Lecithin.

**Table 3:** Composition of hydrogels.

International Nomenclature of Cosmetic Ingredients (INCI NAME)	Amount %		
	blank	MTE	MTE-mp
Methylpropanediol, Caprylylglycol, Phenylpropanol	2.5	2.5	2.5
MTE-mp	//	//	6.0
MTE	//	3.0	//
Arginine	0.15	0.15	0.15
Acrylates/C10-30 Alkyl Acrylate Crosspolymer	0.6	0.6	0.6
Aqua	up to 100	up to 100	up to 100

**Table 4:** Components of emulgels.

INCI Name	Amount (%)				
	Blank	MTE	MTE-mp	L-Blank	L-MTE-mp
**Oil phase**					
Cetearyl Alcohol	4	2.5	2.5	2.5	2.5
Caprylic/Capric Triglyceride	1.5	4	4	4	4
Dimethicone	1.5	1.5	1.5	1.5	1.5
Glyceryl Laurate	2	1.5	1.5	1.5	1.5
Steareth-21	3	2	2	2	2
Steareth-2	4	3	3	3	3
PPG-15 Stearyl Ether, BHT	5	4	4	4	4
Butyrospermum Parkii	1	5	5	5	5
Hydrogenated Lecithin	//	//	//	1	1
**Aqueous phase**					
Methylpropanediol, Caprylyl Glycol, Phenylpropanol	2.5	2.5	2.5	2.5	2.5
Aqua	up to 100	up to 100	up to 100	up to 100	Up to 100
Arylates/C10-30 Alkyl Acrylate Crosspolymer	0.15	0.15	0.15	0.15	0.15
MTE	//	3.0	//	//	//
MTE-mp	//	//	6.0	//	6.0
Arginine	0.1	0.1	0.1	0.1	0.1
